# The Effect of Gender Policies on Fertility: The Moderating Role of Education and Normative Context

**DOI:** 10.1007/s10680-015-9356-y

**Published:** 2016-02-15

**Authors:** Pau Baizan, Bruno Arpino, Carlos Eric Delclòs

**Affiliations:** 1 ICREA, Barcelona, Spain; 2 Universitat Pompeu Fabra, 25 Ramon Trias Fargas Street, 08005 Barcelona, Spain

**Keywords:** Fertility, Gender policies, Gender inequality, Gender division of labor, Gender norms, Europe

## Abstract

In this paper, we aim to assess the extent to which individual-level completed fertility varies across contexts characterized by policies supporting different gender division of labor models. We examine key labor market and care policies that shape gender relations in households and in the public domain. We also consider the role of gender norms, which can act as both a moderator and a confounding factor for policy effects. We hypothesize that, by facilitating role compatibility and reducing the gendered costs of childrearing, policies that support gender equality lead to an increase in fertility levels and to a reduction in fertility differentials by the level of education. Using individual-level data from the European Union Survey on Income and Living Conditions for 16 countries, combined with country-level data, we analyze completed fertility through multilevel Poisson’s models. We find that the national level of childcare coverage is positively associated with fertility. Family allowances, prevalence of women’s part-time employment and length of paid leaves were also found to be positively associated with completed fertility, though the associations were not statistically significant. These variables show a significant positive pattern according to education. A high number of average working hours for men are negatively associated with completed fertility and show a strong negative pattern by educational level. The prevalence of gender-egalitarian norms is highly predictive of fertility levels, yet we found no consistent evidence of a weaker association of gender-equality policies in countries where egalitarian values are less prevalent.

## Introduction

Gender systems are increasingly recognized as a critical component in explanations of fertility levels and differentials, in both advanced and less economically advanced societies (Bernhardt 1993; Folbre 1994; Joshi 1998; Rindfuss and Brewster 1996; Mason 1997; McDonald 2000a, b; Neyer et al. 2013; Esping-Andersen and Billari 2015). Different dimensions of the gender system, including divisions of labor, power and norms, have been related to fertility patterns, and several contributions highlight the key role of social institutions and their close relationship with the gender relations models that exist in different societies.

A growing number of empirical studies have complemented this theoretical literature, generally supporting it, but some contradicting and inconclusive results have also been reported. Though several studies have carefully analyzed the influence of the gender division of labor in households as well as the influence of gender attitudes (e.g., Torr and Short 2004; Oláh 2003; Cooke 2004; Brodmann et al. 2007; Mencarini and Tanturri 2004; Mills et al. 2008; Goldscheider et al. 2013), other dimensions of gender relations, such as women’s capacity to form independent households or the relative level of resources and their connection to fertility, have received less attention (Neyer et al. 2013).

Some have also focused on the links between contextual variables and fertility levels. For instance, Myrskyla (2011) contends that the existing positive link between high human development and the total fertility rate is explained by gender-equality levels. Society levels of gender (in)equality have been further explored by Mills (2010), using a variety of macro-level equality indices, with results showing little or no impact on fertility intentions and behavior in European countries. The extensive literature on the impact of family policies on fertility has also provided important insights (see Gauthier 2007; Hoem 2008; Thévenon and Gauthier 2011, for reviews). However, though some elements of the gender perspective have sometimes been taken into account (Neyer and Andersson 2008; Billingsley and Ferrarini 2014), in most cases, this literature is not based on gender theory.

There is a surprising lack of analyses of the fertility effects of “gender policies,” i.e., government programs that support particular gender relations models. These programs include regulations and welfare provisions, which structure the relative roles of families, markets and the state. In this paper, we link gendered perspectives on the welfare state with demographic theories on the influence of gender relations on fertility patterns. More specifically, because labor market institutions and “social care” are crucial in shaping gender relations (Lewis 1998; Gornick and Meyers 2003; Pettit and Hook 2009; Esping-Andersen 2009; Korpi et al. 2013; Keck and Saraceno 2013), we examine whether and how polices that influence the gender division of labor inside and outside the household are associated with completed fertility at the individual level.

It is important to acknowledge that policies are likely to have heterogeneous effects in the population. Differentials by social class, ethnicity or education are seldom analyzed, thus blurring those effects. Here we examine educational level differentials, as they are closely linked to gender-equality values and to the economic and non-economic costs of childbearing (Joshi 1998; McDonald 2000a; Hakim 2000; Hook 2010). We show that the strength of the association between most policy indicators and completed fertility significantly differs depending on the educational level of women in a theoretically interpretable manner, thus providing a useful way of investigating contextual effects. These results provide some clues regarding the changing individual-level correlation between education and fertility, which has been reported in several countries. For example, in Sweden, highly educated women now exhibit higher, or at least not lower, fertility than the less educated (Andersson 2000), and the educational gap has also been reduced across birth cohorts in Norway and the USA (Kravdal and Rindfuss 2008; Shang and Weinberg 2013).

We also consider the influence of gender norms for two reasons. First, the dynamics of gender-equity norms may be associated with policy changes. Second, the two may interact. For instance, Bonoli (2008) argues that gender-equality policies cannot be expected to impact positively on fertility in a traditional society. We therefore examine whether the statistical association between several policies and fertility differs according to the degree of gender egalitarianism prevalent in the society.

The relationship between selected indicators of policies and individual-level fertility is examined using contextual data as well as data from the European Union Survey on Income and Living Conditions (EU-SILC) for women between ages 36 and 44 during the years 2004–2009. Our empirical analyses focus on 16 Western and Southern European countries showing a variety of fertility levels and policy patterns. Lack of comparable contextual data precluded us from including several Central and Eastern European countries in the analyses.

We apply multilevel regression models to explain completed fertility. Our focus on completed fertility is due to its high political and scientific relevance (despite the fact that it is seldom studied), but timing and parity differentials could not be investigated in this paper due to space and data limitations. It should be taken into account that data limitations prevent us from making strong causal statements, and thus, we examine whether different policy and normative contexts are associated with fertility outcomes at the individual level. As we discuss later in the paper, our findings are highly consistent with theories relating gender relations and fertility levels. Furthermore, our results suggest that, in the context of low fertility societies, welfare state support to gender equality is a crucial factor for a recovery of fertility levels.

## Gender Equality and Fertility: The Role of Policies

Perhaps the most compelling and influential arguments linking gender relations to fertility are those made by McDonald (2000a, b, 2006). He explains that the well-known contrast in fertility levels between, on the one hand, Northwestern European countries, the USA and Australia, where near-replacement levels are reached, and on the other hand, the below-replacement levels predominant in Central, Eastern and Southern Europe and Japan, is a result of the levels of gender equity that exist in the different institutional spheres in each of these groups of countries. While the level of gender equity in access to education and the labor market (i.e., institutions which deal with people as individuals) has reached relatively high levels in most advanced countries, the levels in institutions which deal with people as members of families, such as industrial relations (the terms and conditions of employment), services, government transfers and taxation, and within the family itself, are highly variable among these countries. In countries where the change toward more equality in family-oriented institutions has been slow, the psychic and economic opportunity costs of children increase, leaving women with stark choices between children and employment. As the level of incoherence between social and economic institutions and the dimension of gender equity increases, this leads to some women having fewer children than they would like to have, and very low fertility. McDonald highlights the importance of cultural values and idealized family morality in slowing down changes in family-oriented institutions, arguing that gender equity in both individual- and family-oriented institutions is necessary for fertility to rise—otherwise the costs of fertility fall disproportionally on women.

### Welfare Regimes Perspective

McDonald’s theory links fertility levels and patterns with the functioning of welfare regime institutions. The welfare regime approach provides a suitable framework for understanding relationships between institutions and is the central theoretical framework for international comparative social-policy research (Esping-Andersen 1990, 1999; Mayer 2001). A welfare regime “can be defined as the combined, interdependent way in which welfare is produced and allocated between state, market, and family” (Esping-Andersen 1999, p. 35). The well-known classification of social-democratic, liberal and conservative regimes rests on the relative weight of each of these three institutions, which in turn is rooted on the longstanding cultural ideas that are prevalent in each society. Thus, the principles of egalitarianism and generous state-provided income protection and social services are typical of social-democratic countries, while the ideas of individual responsibility and *laissez*-*faire* state policy characterize liberal regimes. Conservative regimes boost the principles of status segmentation and familism (i.e., the persistence of gender specialization in household work).

Although this classification was initially devised to explain social class stratification, it has also provided an adequate framework for analyzing gender inequality. The gender and care dimensions have been progressively incorporated into analyses of welfare regimes (Orloff 1993; Lewis 1992; Esping-Andersen 1999). Several alternative classifications have been proposed, focusing on particular dimensions such as care or working time, with different degrees of overlap with the original “Three Worlds” typology (Bettio and Plantenga 2004; Hook 2010). However, what is relevant for us here is the emphasis of the welfare regime approach on the functioning of institutions, rather than particular typologies. For instance, focusing on households directs analysis to the paid and unpaid work that needs to be done by household members, its gender distribution and how policies can affect them. This perspective also pays attention to the relative weight of each institution, thus providing a proper context in which to understand the role of particular polices and its complementarities or contradictions with respect to other policies and other institutions.

Each welfare regime type is associated with distinct patterns of social class, life course and intergenerational risks, which are managed in different ways and with different distributional consequences by the market, the state and households (Esping-Andersen 1999). As highlighted by feminist literature on the welfare state, gender-specific risks can also be added to this list. Systematic variations by regime type can be found in the so-called mother penalty associated with childbirth, the extent to which divorce or lone motherhood is associated with poverty, gender differentials in unemployment and labor force participation, and the degree of marginalization in relation to the social security system (Sigle-Rushton and Waldfogel 2004). Furthermore, these patterns are related to women’s capability to form independent households and to make genuine choices about care and childbearing (Orloff 1993; Neyer et al. 2013). State policies are particularly important in understanding these patterns, since they have a critical role in structuring and shaping institutions. Here we highlight two dimensions of these policies that are particularly relevant for gender equality and its relationship with childbearing: care policies and labor market policies.

### Care Policies

Two policy strategies have been adopted to “defamilialize” childcare work.^1^ The first is based on the state provision of early childhood education and care services. The second is based instead on creating the conditions for market provision, through labor market and childcare regulations, often supplemented by subsidies and tax deductions. There are different policy mixes in European countries, which lead to different coverage rates and different outcomes in terms of equality of access and the degree of heterogeneity in the quality of care (Bettio and Plantenga 2004; Plantenga et al. 2008). Of course, family care has remained substantial and, to the extent that women are still the main providers of it, a strong reliance on household care constrains their labor market participation. Furthermore, family care activities have a crucial role in child development (Waldfogel et al. 2002) and are likely to be a central motivator for fertility behavior. Thus, it is relevant to consider how policies support (or hinder) care activities at home as well as the way they shape labor market participation.

### Labor Market Policies

Though gender discrimination in the labor market has been reduced since the 1960s, a substantial degree of vertical and horizontal segregation persists. Labor market reforms have often pursued the labor market “activation” of women (Lewis 2002; Cipollone et al. 2013). At the same time, there is a huge variety among European countries in the extent to which labor market organization takes into account that individuals have family responsibilities. In this respect, working-time arrangements set by national regulations and collective agreements are of particular importance, since they both establish how much time is available for caregiving and constrain parents’ ability to share care among them. Long standard work weeks have been shown to lead to a reduced involvement of fathers in childcare and housework (Hook 2010; Baizan et al. 2014) and are related to low proportions of women in full-time jobs (Rubery et al. 1998; Williams 2000). Part-time work has been promoted by several governments as a response to the work/family conflict; however, it remains almost exclusively a female response that does not question the women’s primary responsibility for childcare, while involving significant income differentials by gender.

Labor market policies also include more explicit regulations supporting the familialization of care with time and transfers. There is a wide variety in leave provisions in Europe, with different consequences for gender equality in the labor market and care work (Sigle-Rushton and Waldfogel 2004; Rubery et al. 1998; Hook 2010). These provisions imply a “modernization” of the care arrangement, in the sense that they make full- or part-time dedication to childcare a temporary stage in women’s labor market trajectories (Pfau-Effinger 2005), significantly reducing the costs of childrearing. However, leave provisions are highly gendered, and fathers’ take-up of leave time has remained marginal, except in the Nordic countries (Duvander et al. 2010). Women’s decisions to enter or stay in the labor market are further shaped by policies regarding benefits and taxes in connection to children, which may provide incentives or disincentives for participation (Del Boca et al. 2009; Cipollone et al. 2013).

The abovementioned policy arrangements reflect the combined effects of many different and often contradictory forces. According to Korpi (2000a, b), welfare policies are the outcome of political struggle. In Western Europe, the main actors involved in that struggle have been political parties, unions, churches and women’s movements. This author highlights the role of confessional parties in supporting family care and maintaining gender differentials, as opposed to social-democratic and liberal parties. Policy inertia and entrenched interests may lead to contradicting layers of policy arrangements.

### The Impact of Norms

It is necessary to bear in mind that institutions are always embedded in broader social contexts that include citizens’ attitudes, norms and values, which may support or counteract policy outcomes. In a recent work, Esping-Andersen and Billari (2015) linked the evolution of gender-equality norms with fertility developments. The authors depict the gender-equality “revolution” as a process of diffusion of new norms, in which a relatively high-fertility/low-gender-equality equilibrium is broken by a transition toward a more egalitarian society, which in its first steps brings about a decline in fertility rates. Then, a recovery of fertility is expected, when the gender-equity revolution is completed. Arpino et al. (2015) provide empirical evidence in favor of a *U*-shaped relationship between changes over time in gender-equitable attitudes and fertility at the country level. Key discontinuities that trigger the start of changes in the long-term trends are brought about by increases in the education of women, improving household technologies and modern contraception.^2^ Some institutional characteristics of societies (such as trust or stratification) may speed up or hinder that process.

Though the evolution of gender norms in a given society can be theorized along a continuum, it remains a challenge to explain the strong diversity that exists among European countries. In this respect, Pfau-Effinger proposes a classification with five cultural models about the family and the way it is related with gender and care: the housewife model of the male breadwinner family; the family economy model;^3^ the male breadwinner/female part-time carer model; the dual-breadwinner/external carer model; and the dual-breadwinner/dual-carer model (Pfau-Effinger 2004; Crompton 1999). The prevalence of each of these models is highly variable across European countries, and it can be assumed that they are closely related to the particular policies that support them. Nevertheless, though they may not exactly parallel state welfare policy packages, gender ideologies tend to be related to them (Gornick and Meyers 2003). The evolution of gender norms seems to be closely related to variations in the institutional context of support for working parents (Anxo et al. 2007): For example, countries with greater childcare coverage tend to have higher maternal labor force participation rates and less rigid gender roles. We must also highlight the role of institutional arrangements and policies not only in creating opportunity structures for making family formation decisions, but also in influencing family values in a dynamic way (Bowles 1998; Jakee and Sun 2001). Moreover, social interaction can lead to a multiplier effect of policies (Fent et al. 2013). Thus, family policies, gender norms and labor market conditions are interrelated, and this must be taken into account when studying the relationship between policies and fertility.

The variety of family and gender arrangements in European countries underlines that institutional change does not follow one single path. It also suggests the existence of “unstable equilibriums” (Esping-Andersen and Billari 2015), in which the existence of policies supporting a particular model may tip the balance toward it, thus promoting the prevalence of that model in the population. At the same time, although there are several examples of changes in particular policies, the overall policy frameworks tend to be highly stable. Many studies emphasize the long-standing differences in social institutions and their typically slow and path-dependent evolution (McNicoll 1994; Mayer 2001).

### The Gender Transition

Since the 1970s, advanced societies have been moving away from the assumption of the breadwinner–homemaker family in favor of more gender equality. Cross-national variations are substantial, but it is possible to summarize overall *policy* trends in four “ideal–typical” stages starting from the situation in the 1960s, when all countries were dominated by policies supporting the male breadwinner model. In the first stage of the gender transition, women enter the labor market, but there is no institutional adaptation to women’s participation. This often leads to a “double-shift” scenario with declining fertility.

In the second stage, there is some institutional adaptation to women’s new roles. Barriers to women’s labor market participation are gradually removed, so that they can reap the benefits from it. Policies may include the promotion of part-time jobs, the introduction of (paid) parental leaves and the expansion of formal childcare provision. The goal has often been to accommodate or promote women’s employment, resulting in the creation of an adult worker model for both genders (Orloff 2002). As time-use analyses have shown, at this stage, men’s roles have only marginally changed (Kan et al. 2011), precluding gender equality in both the labor market and the home. These changes amount to a modification of the traditional male model of work and welfare and its generalization to women (Lewis 2002).

In the third stage, institutional adaptation begins to focus more on men’s roles. Men take an increasing responsibility for care and domestic work, and their labor market involvement starts to change, for instance, by increasing their use of paternity leave. Policies supporting these changes include the promotion of fathers’ leaves and adapting the work environment to the growing caring role of men—such as shorter working hours or more flexible time schedules. Even if gender-equality values are highly prevalent, it is not yet clear what economic or social incentives men may have to adopt what have traditionally been women’s roles (González et al. 2009).

The fourth stage involves a fully egalitarian model, in which both men and women are workers and carers to a similar extent (the “dual-earner/dual-caregiver” society). No society has reached this stage yet. Increasing rates of female employment have narrowed the gender gap in labor force participation, but gender differences persist in such areas as career breaks, occupational segregation, working hours or pay. And although men’s engagement in domestic work and caregiving has increased in many countries, nowhere does it match women’s influx into paid employment. In reality, the stages overlap, and the timing of changes is likely to be particularly important for fertility outcomes. If change comes late but very rapidly, as in Southern Europe, this might provoke very low fertility. If institutions begin to adapt early, as in Scandinavia, then a higher fertility level is easier to sustain.

## The Differential Impact of Gender Policy Models: Hypotheses

In the theoretical framework sketched above, gender-equality polices have the role of providing concrete resources and opportunities to women and families as well as the role of influencing norms in a society. These policies mitigate the gendered costs of childbearing by supporting particular models of families and gender relations. Thus, the institutional context, including policies, helps to determine the structure of costs and rewards of fertility. To the extent that particular policies reduce the direct and especially the indirect economic costs of childbearing, they can have a positive effect on fertility (Hotz et al. 1997; DiPrete et al. 2003). Non-economic costs associated with cultural norms and individuals’ values are also likely to be relevant for fertility. The reduction in women’s labor force participation linked to having children involves costs such as detachment from the paid labor force, the loss of social networks, the loss of skills, the loss of social status, the loss of self-esteem and especially the loss of gender equality in the couple (Joshi 1998; McDonald 2000a). Again, gender policies can reduce these costs, leading to an increase in fertility. Therefore, in the context of countries with high levels of women’s education and where a large fraction of women has joined the labor force (i.e., all the countries considered in this study), it can be hypothesized that *public policies that support egalitarian gender relations have an overall positive effect on fertility* (Hypothesis 1). Conversely, policies centered on the male breadwinner model are likely to be counter productive for rising fertility levels. In order to empirically examine this hypothesis, we use several indicators of policies that are expected to reflect key dimensions of the effect of the gender system on fertility (see the section on data for details on the indicators used in the analyses). In particular, we focus on “work-family reconciliation” polices, for both men and women, in the areas of working-time regulations and childcare (including policies that provide time or money to care). Of course, we acknowledge that many other polices have a potential impact on fertility or on gender relations. For instance, health and education subsidies decrease the cost of children for parents, and elderly care policies may influence women’s involvement in paid work, etc. Nevertheless, here we focus on polices that directly influence the gender division of labor for parents, which is considered a critical element by the theoretical arguments cited above.

Though policy factors provide a common context for all individuals, they may not affect all of them in the same way. Societies provide particular structures of incentives and costs of having children that apply to some individuals (or couples) more than others. In particular, the decline in women’s status that is often observed upon the birth of a child is closely related to her employment situation and educational level. The economic and psychic costs of having children tend to be particularly high for highly educated women if the combination of parenthood and paid work is difficult. As a consequence, fertility drops are generally severe for them in the early stages of the gender transition, while educational differentials tend to disappear, or even reverse, in more advanced stages. Opportunity costs, i.e., the loss of income related to time out of the labor force to care for children, have been rightly highlighted in the literature, while the direct economic costs seem to be relatively less important for women with high market productivity (Hotz et al. 1997). Yet, as the educational level of women increases, the non-economic costs of a reduction in labor force participation associated with having children are likely to become more important. Education is also associated with a higher prevalence of gender-egalitarian values for both women and men, such that these costs are likely to be more relevant for highly educated individuals. It is, for example, widely documented that higher-educated couples are far more disposed toward gender egalitarianism (Coltrane 2000). Less-educated women not only face lower opportunity costs of interrupting careers but are also more likely to find themselves in precarious labor market situations, making conventional gender roles appear more attractive. Role specialization is also likely to be further reinforced by unequal bargaining positions and by social approval expectations, especially among family members. Finally, it should be taken into account that education is associated with higher incomes and employment security. A positive “income effect” on fertility can be expected from this association, premised on the existence of policies favoring the compatibility between care and paid work.

Overall, we would expect that welfare states with a strong gender-egalitarian profile should help narrow the fertility gap between higher- and lower-educated women. Thus, our second hypothesis is that *the higher the level of gender equality prevalent in the welfare regime institutions is, the lower the negative association between women’s level of education and fertility will be* (Hypothesis 2). This logic is applied to specific contextual factors or policies, to the extent that they give support to a particular gender arrangement. For example, *childcare services* favor the combination of work and parenthood and therefore should have a generally positive effect on fertility, following our first hypothesis. Additionally, we hypothesize that the availability of formal childcare should have a stronger positive effect on the fertility of highly educated women relative to the low-educated women. Improved access to childcare means that wage-dependant opportunity costs are substituted by childcare costs, which are generally less dependent on income (Ermisch 1989). Since better-educated women have a higher involvement in paid work and higher wages, they will benefit more from the reduction in opportunity costs resulting from an increased availability of formal childcare.

As suggested above, a high number of standard *working hours for men* can be considered a barrier to their involvement in childcare and unpaid work, while labor market policies that enable fathers to reallocate time for caregiving support dual-earner–caregiver families. We therefore expect that this indicator negatively affects fertility, especially for highly educated mothers, since it reinforces gender role specialization.

The availability of *part*-*time work* facilitates the combination of paid and unpaid work. Thus, a generally positive effect on fertility can be expected. In the early stages of the gender transition, a large fraction of women may seek this arrangement, except among lower-educated women, which may tend to prefer a full-time housewife role. However, women’s part-time work also favors a gender specialization model in which women are secondary earners and main caregivers, which may not be attractive to highly educated women. Furthermore, part-time work characteristics differ considerably between countries. In Southern Europe, it is often associated with precarious working conditions, while in the Nordic countries and the Netherlands, it is more compatible with career jobs, potentially enhancing the ability of more educated women to reach their fertility goals. These contradicting influences may weaken the expected positive effect of this variable.

In principle, the existence of *paid parental leave* for women can enhance the compatibility of paid and unpaid work, potentially having a pro-natalist effect. In fact, in all countries studied, there is a minimum paid leave period with a guaranteed return to the job. However, the effect of this variable on fertility may not be clear-cut, since very long leaves (i.e., more than a year) tend to negatively affect labor force attachment. Additionally, short leaves have been associated with poorer health and developmental outcomes for children (Waldfogel et al. 2002). Furthermore, leave policies focused on women reinforce their caregiver role, while decreasing the caregiver role of men. Unfortunately, a measure of father’s leave use is not available for all the countries studied, but it would be a powerful indicator of an evolution toward a gender-equal arrangement. Well-paid leaves should have a positive effect on fertility, especially for highly educated women. In fact, based on economic theory, a higher pay rate (e.g., 100 % instead of 80 % of the salary) would imply a reduction in the opportunity cost of childbearing and thus may be expected to have a positive effect on fertility (Gauthier and Hatzius 1997).

*Child benefits and tax deductions* may only be aimed at reducing the direct cost of children and can be designed in a way that does not create disincentives for women’s labor force participation (Gustafsson and Stafford 1994). However, they often reflect the logic of the conventional male breadwinner model (Orloff 2002). On the whole, these kinds of policies can be hypothesized to have a higher positive impact on the fertility levels of low-educated women, because financial incentives are likely to represent a higher proportion of the cost of children for low-income couples.

If the effects of policies can vary according to the prevailing *gender norms* existing in different countries, this suggests the convenience of including a measure of them in the analyses. Furthermore, as outlined above, the effects of gender norms and policies influence each other, reinforcing their effects over time. Therefore, *we expect that the effect of policies favoring the combination of work and parenthood will be stronger the more gender*-*equal a society is* (Hypothesis 3), i.e., in statistical terms, we expect a positive interaction between policies and macro-level gender equality. By the same token, policy measures that conflict with a gender-equality model or favor gender role specialization, such as short-paid parental leaves, certain types of family benefits, or even part-time work, could be negatively associated with fertility levels when combined with a high proportion of adherence to gender-egalitarian norms in the population.

## Data and Descriptive Statistics

We use the EU-SILC longitudinal data for the years 2004–2009 for 16 Western and Southern European countries for which we also have contextual information. We restrict our analyses to the most recent observation of women aged 36–44 years, yielding a working sample of 69,213 women (the number of women per country ranges from 2326 to 13,871). The dependent variable used in all the analyses is the total number of own children living in the same household as the mother at the time of interview. This variable approximates the completed fertility of women. In Table 1, we compare the average number of children per women as estimated with EU-SILC data with data on completed fertility from the Human Fertility Database (HFD 2014). Women in our sample were born between 1960 and 1973, but completed fertility data from HFD are available for the cohorts born between 1935 and 1968. Moreover, data are not available for all countries in our dataset. However, for ten countries, we can compare data for the cohort born around 1966 which corresponds to approximately the central year in which women in our sample were born. Table 1 shows that, usually, estimates from EU-SILC are very close to completed fertility data from HFD. Nonetheless, our goal is not to describe completed fertility at the country level but to estimate associations between number of children at the individual level and individual- and country-level independent variables.

**Table 1:** Completed fertility by country: EU-SILC estimates compared to HFD data

Country	EU-SILC	EU-SILC	EU-SILC	HFD
	All years	1964–1968	1966	1966
Austria	1.63	1.64	1.60	1.64
Belgium	1.71	1.78	1.90	…
Denmark	1.88	1.90	1.85	…
Finland	1.95	1.95	1.94	1.92
France	1.73	1.70	1.68	2.02
Germany	1.58	1.62	1.59	1.52
Greece	1.64	1.67	1.75	…
Ireland	2.03	2.06	2.22	…
Italy	1.38	1.38	1.42	…
Luxembourg	1.71	1.67	1.58	…
Netherlands	1.80	1.82	1.82	1.78
Norway	2.04	2.09	2.12	2.07
Portugal	1.66	1.66	1.61	1.82
Spain	1.53	1.54	1.58	…
Sweden	2.02	2.06	2.08	2.00
UK	1.67	1.70	1.74	1.90

Our working sample from EU-SILC data includes women born between 1960 and 1973. About 50 % of the sample was born in the years 1964–1968. For some countries, the Human Fertility Database (HFD 2014) provides data on completed fertility for cohorts born between 1935 and 1968. We report data for the 1966 cohort or if not available (Finland, Netherlands, Norway) for the 1965

The explanatory variables include, firstly, the respondent’s age at the time of the survey and the highest educational level attained. We have coded education into three categories: some secondary education and less, completed secondary and/or post-secondary non-tertiary education (the reference category that includes the range from upper secondary studies to post-secondary education that cannot be regarded as university-level studies) and university studies or higher.

We complement the micro-level data with country-level data on policies, labor market conditions and norms. In particular, we consider data on family benefits, leaves, availability of formal childcare, labor market conditions and the prevalence of gender-equitable attitudes (as an indicator of norms). We use contextual information reflecting the situation in the mid-1990s (1992–1998), when most of the women in our sample had their children. When data are not available for this period, we consider the oldest data available. In any case, variations in country-specific indicators over time are very limited. The values of our macro-level indicators are reported in Table 2.

**Table 2:** Macro-indicators by country, average values in the period 1992–1998 or in the closest available year to 1998

Country	Family allowances (1992–1998)	Weighted leave weeks (1992–1998)	Childcare coverage (2004)	Childcare usage (2004)	Men working hours (1992–1998)	%Women on part-time (1992–1998)	Gender-egalitarian norms (1999)
Austria	98.54	49.64	9.00	4.00	41.13	21.85	61.85
Belgium	128.72	24.17	34.00	42.00	40.61	30.35	68.55
Denmark	65.05	32.23	56.00	73.00	38.33	26.28	91.60
Finland	100.01	79.01	21.00	27.00	40.55	11.74	87.07
France	110.93	76.17	43.00	32.00	41.37	24.49	72.65
Germany	79.57	39.71	10.00	16.00	41.06	29.16	73.03
Greece	14.56	7.79	7.00	7.00	45.35	13.50	80.10
Ireland	32.89	9.80	15.00	20.00	44.86	26.73	74.50
Italy	79.61	25.00	11.00	25.00	41.34	21.06	65.00
Luxembourg	150.89	42.00	14.00	22.00	41.68	25.74	73.80
Netherlands	79.50	15.43	15.00	40.00	37.93	54.33	81.10
Norway	100.22	41.19	37.00	33.00	38.52	37.56	84.85
Portugal	21.21	13.53	19.00	30.00	44.11	15.16	68.40
Spain	24.55	14.86	17.00	39.00	42.17	15.18	75.90
Sweden	74.14	49.95	50.00	53.00	39.38	23.72	94.95
UK	54.51	7.79	26.00	29.00	44.27	41.04	73.17

For family allowances, weighted leave weeks, men working hours and percentage of women working part-time, we report the average value for the period 1992–1998. Data on men working hours are only available for 1995–1998 for Austria, Finland, Norway and Sweden. For Austria, data on the percentage of women working part-time are only available for 1995–1998. For the other variables, data refer to a specific year (the closest available to 1998): 2004 for childcare coverage and usage and 1999 for gender-egalitarian values. Data on family allowances and weighted leave weeks are taken from the Comparative Family Policy Database (Gauthier 2011); childcare coverage and usage are taken from the Multilinks database (Keck et al. 2009); men working hours and the percentage of women working part-time are obtained from OECD (2014); gender-egalitarian norms are based on our calculations on WVS–EVS data

Regarding family benefits, we include the following indicator:

- Monthly family allowances in US dollars (PPP adjusted) for the second child (*family allowances*), years 1992–1998. Values are divided by 1000 when using the variable in the regression models.

We also tested for allowances for the first and third child, but results were very similar to those reported. We also considered another indicator for tax and benefit transfers. For the sake of brevity, we do not present the regression results using this indicator, as these were similar to those reported here for monthly family allowance.

Measuring leave benefits is a hard task. There are important cross-country variations regarding overall length, level of compensation and eligibility criteria of maternity and parental leave (Ray et al. 2010; Wall 2007). Saraceno and Keck (2009) note that some countries (e.g., France) offer both very long and well-compensated parental leave. Other countries, like Spain, offer quite long leaves but pay only a short period of them, while still others, such as Greece, offer a leave that is comparatively short. Moreover, while almost all developed countries include a period of maternity leave, some countries also offer parental and/or childcare leaves, i.e., optional leave periods available after the period covered by the maternity leave scheme and usually not restricted to mothers. Similar to Gornick and Meyers (2003), our indicator takes into account these three types of leave, their duration and level of compensation. Specifically, we use the following indicator:

- Sum of weeks of maternity, paternity and childcare leave weighted by the level of cash benefits paid during each type of leave (measured by the percent of female wages in manufacturing, Gauthier 2011). The resulting indicator can be interpreted as the total equivalent number of leave weeks paid at 100 % (considering average female salaries) (*weighted leave weeks*), years 1992–1998.

Data on family benefits and leaves are taken from the Comparative Family Policy Database (see Gauthier 2011 for details on the indicators and sources of data).

To measure the availability of formal childcare, we use the childcare coverage measure contained in the Multilinks database (Saraceno and Keck 2009) and defined as:

- Number of places in public (or publicly subsidized) childcare facilities as a share of the number of children aged 0–2 years (*childcare coverage*), year 2004.

When analyzing the effect of policies, childcare coverage is a more meaningful indicator to use than indicators of the *usage* of childcare services (for a discussion see Saraceno and Keck 2009). However, as data on usage are frequently employed, we also considered the following indicator:

- Children aged 0–2 years cared for in formal childcare arrangements as a percentage of all children in the same age group (*childcare usage*), year 2004.

Data on childcare usage are also reported in the Multilinks database. These data, made available by Eurostat, are obtained by the aggregation of micro-level data from EU-SILC. As can be seen in Table 2, coverage and usage for some countries differ quite substantially. According to Saraceno and Keck (2009), this is mainly due to the fact that coverage only refers to public or publicly subsidized services, while EU-SILC data on usage also include private formal childcare.

To capture labor market conditions, we include two indicators from OECD data (2014):

- Standard number of working hours per week for men (*men working hours*), years 1992–1998.
- Share of women working part-time out of the total number of employed women (*women on part*-*time*), years 1992–1998. Part-time is defined as less than 30-week-usual hours worked in the main job.

Finally, in order to measure the spread of gender-egalitarian norms, we use data from the World Values Survey (WVS) and the European Values Study (EVS). We include data from the oldest wave for which our indicator is available (1999). Our measure of gender-egalitarian norms is based on a widely used item in gender studies (e.g., Arpino and Tavares 2013; Arpino et al. 2015; Seguino 2007): “When jobs are scarce, men should have more right to a job than women.” The questionnaire offers three possible answers: (1) “agree,” (2) “disagree” and (3) “neither.” We recode the variable into a binary response: 0 is “agree” or “neither” and 1 is “disagree” and calculate the percentage at the country level.

We decided to use only one question instead of an index summarizing more than one item because the chosen indicator clearly measures views toward gender roles in the labor market, making it the most relevant to an analysis such as ours, which focuses on the contextual factors facilitating the reconciliation of motherhood and a career. As argued by Arpino et al. (2015), this item has both a high theoretical and empirical validity. Among all the items available in the WVS–EVS data, the one we use shows the highest adherence to the concept of gender equity because it is the only question that clearly puts men and women into juxtaposition in terms of the adequacy of their respective gender roles. For these reasons, it is the item that more closely resembles the ones proposed by McDonald (2000b) to measures the share of people who agree with gender-equity values in a given country. However, it is important to keep in mind that it focuses only on the labor market dimension of gender roles. For the sake of simplicity, from this point on, we will refer to this measure as the gender-equality indicator.

## Methods

Since our dependent variable is a count variable (number of children), we employ Poisson’s regression models to estimate the association between individual and contextual independent variables and fertility.^4^ In order to take into account that our dataset has a hierarchical structure with women clustered in countries, we use multilevel (random effects) Poisson’s regression models to adjust for intra-country correlation (Skrondal and Rabe-Hesketh 2004).

In a multilevel Poisson’s regression model, we basically model the probability that the outcome variable takes a given value (count) as a function of a set of covariates and random effects:

1 $$ P(y_{ij} = y) = \exp [ - ({\mathbf{x}}_{{{\mathbf{ij}}}} {\varvec{\upbeta}} + u_{j} )]({\mathbf{x}}_{{{\mathbf{ij}}}} {\varvec{\upbeta}} + u_{j} )^{y} /y! $$

where the subscript *ij* indicates a woman belonging to country *j*, *y*_*ij*_ denotes the responses (number of children for woman *ij*; *y* = 0, 1, 2,…), *x*_*ij*_ represents the independent variables with (fixed) coefficients *β* and *u*_*j*_ represents country-level random effects (“random intercepts”). For each country, *u*_*j*_ is a realization from a normal variable with mean 0 and standard deviation to be estimated. Independent variables can be included at the both individual and country level. Multilevel models can handle unbalanced data structures characterized by different cluster sizes (Skrondal and Rabe-Hesketh 2004), as in our dataset where sample sizes across countries vary. In this case, each cluster automatically contributes to the log likelihood with a weight proportional to its size.

One limitation for the application of a multilevel model in our case is the limited number of countries available in our dataset (16). In fact, multilevel modeling requires a sufficient sample size at each level of the hierarchy. Simulation studies, as summarized in the literature review by McNeish and Stapleton (2014), have shown that with a limited number of clusters (i.e., countries in our case), estimation of variance components (i.e., the variance of random errors at the different levels) is particularly problematic. However, our focus is on the point estimate of “fixed effects” (beta coefficients) to interpret the association between number of children and individual- and country-level predictors. Simulation studies have found that in this case, the use of multilevel models with a small number of clusters, even as few as ten, is less problematic (e.g., Paccagnella 2011).^5^ Moreover, within each country, we have large samples (i.e., the cluster sizes are high) and this is important for avoiding biased point estimates (Austin 2010). In any case, caution is needed in the interpretation of our analyses because the small number of clusters can affect the estimation of standard errors.

All models have been estimated using the command *meqrpoisson* in Stata13. In all models considered, we controlled for age and education. Education was entered as an individual-level covariate. To take into account that the amount of exposure time over which the dependent variable is observed for each women in the sample varies (our sample include women aged 36–44 years), we included the variable age in the *exposure* option. In this way, the estimates are adjusted for the length of time a woman was at risk of having children even though the coefficient of the variable age is not estimated and therefore will not be reported in the tables [log(age) is included in the fixed-effects portion of the model with the coefficient constrained to be 1]. Using age as a covariate yielded results similar to those presented here.

To test our hypotheses, the country-level indicators described in the previous section are also considered as independent variables together with individual controls. A key advantage of using multilevel models in our analysis is that they recognize that the relevant sample size at the country level for estimating coefficients of the country-level variables is the number of countries in the dataset and not the overall sample size. However, having a low number of countries implies that we have limited degrees of freedom at the country level, limiting the number of country-level variables we can simultaneously enter into the model. Moreover, our country-level variables show a quite high degree of correlation (see Table 6). Therefore, to avoid problems of multicollinearity, and as is typically done in cross-national multilevel studies characterized by a low number of countries (see e.g., Aassve et al. 2013), we entered our country-level variables one by one.

Some of our hypotheses involve an interaction between women’s education and macro-level indicators or an interaction between macro-level indicators. To ease interpretation of results, model estimates reported in the tables are complemented by graphs reporting the predicted number of children for different levels and combinations of independent variables. We also report 95 % confidence intervals for pair-wise comparisons. These intervals are centered on the predictions and have lengths equal to 2 × 1.39 × standard errors. As showed by Goldstein and Healy (1995), this is necessary in order to have an average level of 5 % for the Type I error probability in pair-wise comparisons of a group of means. Therefore, in the figures, an overlap in terms of the confidence intervals indicates that predictions are not significantly different, whereas non-overlap reflects that they are.

## Results

We start by presenting the estimates from a series of two-level Poisson’s regression models in Table 3. Model 1 only includes educational levels as an independent variable, while Models 2–7 also add our country-level variables one at time. As explained in the previous section, all models control for age effects. We can see that the estimated variance of the country-level random effect (last row of Tables 3, 4, 5) is always statistically significant, meaning that there is substantial variation across countries in the average number of children and that a multilevel model is needed.

**Table 3:** Estimates of a series of two-level Poisson’s regression models for completed fertility as function of women’s education and macro-variables

Variables	Models						
	(1)	(2)	(3)	(4)	(5)	(6)	(7)
Educational level							
Low	0.094***	0.094***	0.094***	0.094***	0.094***	0.094***	0.094***
Medium (ref.)							
High	−0.041***	−0.041***	−0.041***	−0.041***	−0.041***	−0.041***	−0.041***
Family allowances		0.484					
Weighted leave weeks			0.002				
Childcare coverage				0.004**			
Men working hours					−0.019		
Women share part-time						0.003	
Gender-egalitarian norms							0.009***
Constant	−3.163***	−3.200***	−3.219***	−3.268***	−2.385***	−3.239***	−3.879***
Var(country)	−2.157***	−2.170***	−2.211***	−2.348***	−2.227***	−2.195***	−2.535***

Var(country) indicates the variance of the random effect at the second level (country)

* *p* < 0.05; ** *p* < 0.01; *** *p* < 0.001

**Table 4:** Estimates of a series of two-level Poisson’s regression models for completed fertility as function of women’s education, macro-variables and their interaction

Variables	Models					
	(1)	(2)	(3)	(4)	(5)	(6)
Educational level						
Low	0.120***	0.126***	0.120***	−0.594***	0.167***	0.413***
Medium (Ref.)						
High	−0.081***	−0.049***	−0.092***	0.376**	−0.050**	−0.260***
Family allowances	0.483					
*×Low education*	−0.404^+^					
*×High education*	0.544*					
Weighted leave weeks		0.002				
*×Low education*		−0.001**				
*×High education*		0.000				
Childcare coverage			0.004*			
*×Low education*			−0.001*			
*×High education*			0.002***			
Men working hours				−0.020^+^		
*×Low education*				0.016***		
*×High education*				−0.010**		
Women share part-time					0.003	
*×Low education*					−0.003***	
*×High education*					0.000	
Gender-egalitarian norms						0.009***
*×Low education*						−0.004***
*×High education*						0.003***
Constant	−3.201***	−3.224***	−3.259***	−2.331***	−3.254***	−3.879***
Var(country)	−2.172***	−2.211***	−2.339***	−2.235***	−2.178***	−2.525***

In each model, we include education levels (medium is the reference), one of the macro-variable at time and its interaction with education (in italic). Var(country) indicates the variance of the random effect at the second level (country)

^+^ *p* < 0.10; * *p* < 0.05; ** *p* < 0.01; *** *p* < 0.001

**Table 5:** Estimates of a series of two-level Poisson’s regression models for completed fertility as function of women’s education, macro-variables and interaction between gender-equality norms and policy variables

Variables	Models				
	(1)	(2)	(3)	(4)	(5)
Educational level					
Low	0.094***	0.094***	0.094***	0.094***	0.095***
Medium (ref.)					
High	−0.041***	−0.041***	−0.041***	−0.041***	−0.041***
Gender-equality norms	0.009***	0.009***	0.009**	0.008**	0.009***
Family allowances	0.755				
*×Gender equality*	0.077				
Weighted leave weeks		0.001			
*×Gender equality*		0.000			
Childcare coverage			0.002		
*×Gender equality*			−0.000		
Men working hours				0.001	
*×Gender equality*				−0.001	
Women part-time work					0.004
*×Gender equality*					−0.000
Constant	−3.177***	−3.177***	−3.169***	−3.183***	−3.179***
Var(country)	−2.610***	−2.566***	−2.592***	−2.544***	−2.611***

In each model, we include education levels (medium is the reference), gender-equality norms, one of the macro-variable at time and its interaction with gender-equality norms (in italic). All macro-level variables are mean centered. Var(country) indicates the variance of the random effect at the second level (country)

^+^ *p* < 0.10; * *p* < 0.05; ** *p* < 0.01; *** *p* < 0.001

**Fig. 1:**
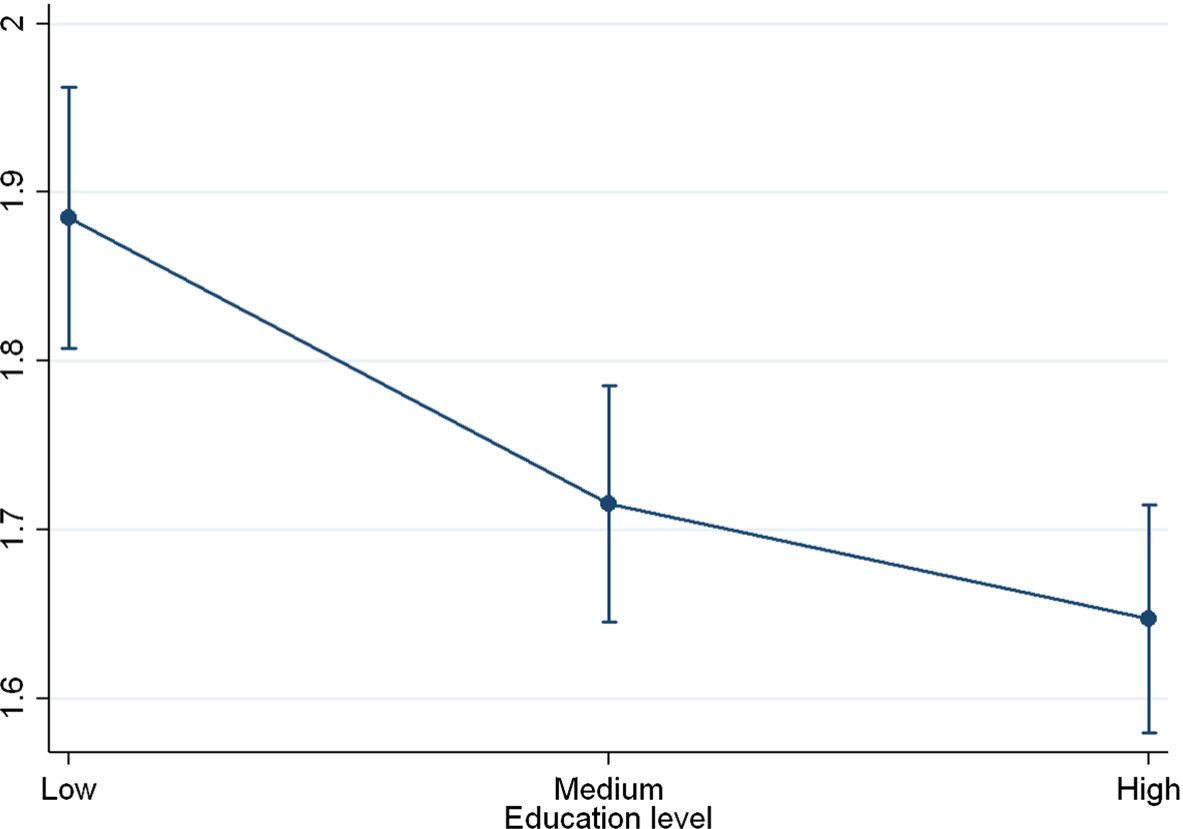
Predicted number of children (*y*-axis) by woman’s educational level (*x*-axis) with 95 % confidence level intervals for pair-wise comparisons. *Note*: Predictions are obtained using estimates from a multilevel Poisson’s regression model (Model 1, Table 3). Confidence intervals are centered on the predictions and have lengths equals to 2 × 1.39 × standard errors to have an average level of 5 % for the Type I error probability in the pair-wise comparisons of a group of means (Goldstein and Healy 1995)

Estimates of Model 1 in Table 3 show that women’s education has a negative and statistically significant relationship with completed fertility. Given the complexity of the Poisson model, to better interpret estimated coefficients, we display the predicted number of children by women’s education level in Fig. 1. This graph shows that the association of education with fertility is substantial, implying that the predicted average number of children for women with lower secondary education or less is about 1.89, for a women with upper secondary education it is 1.71 and for the highly educated it is 1.65. This result is consistent with what is known about cohort educational differentials in completed fertility for the countries we consider (Basten et al. 2013). As noted above, in a few countries (e.g., the Nordic countries), this negative relationship has substantially weakened for recent birth cohorts, as is consistent with a relatively advanced stage in the gender transition.

Results of Models 2–7 provide evidence on the relationship of our country-level indicators with completed fertility. The goal of this analysis is to empirically explore the overall association between fertility and specific policies supporting different gender relations models (Hypothesis 1). In Table 4, we show the interaction of education with each of the contextual variables considered, as a way of testing whether gender-equality policies have a stronger positive association with the fertility of women with a higher level of education (Hypothesis 2).

**Fig. 2:**
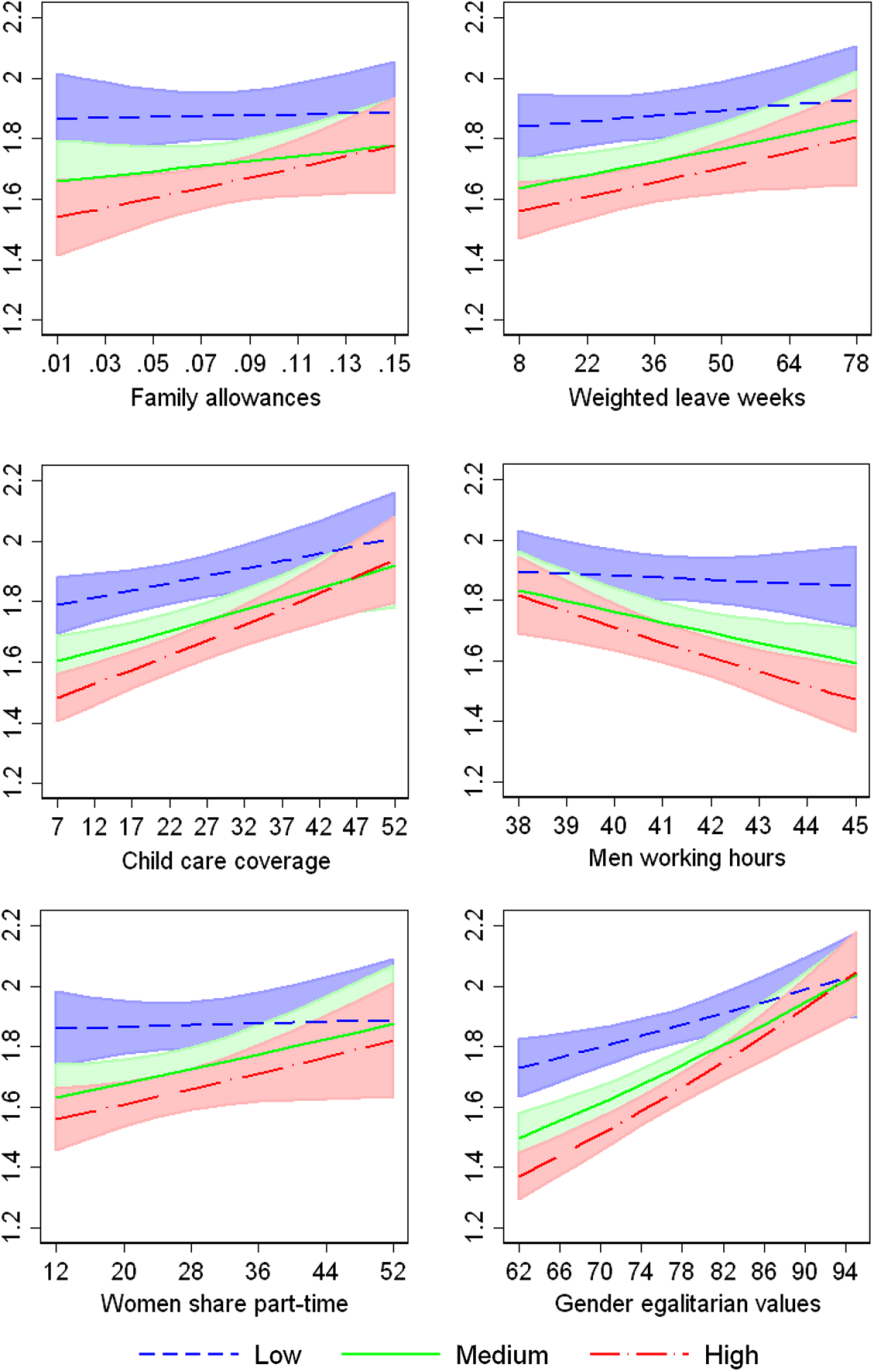
Predicted number of children (*y*-axis) by country-level variables (*x*-axis) and woman’s educational level with 95 % confidence level intervals for pair-wise comparisons. *Note*: Predictions are obtained using estimates from multilevel Poisson’s regression models (Models 1–6, Table 4). Values of country-level variables are ranged over the observed ranged of values. Confidence intervals are centered on the predictions and have lengths equals to 2 × 1.39 × standard errors to have an average level of 5 % for the Type I error probability in the pair-wise comparisons of a group of means (Goldstein and Healy 1995)

Starting with formal childcare coverage for children under 3, an overall significant positive association with fertility is found (Model 4 in Table 3 shows a coefficient of 0.004, with *p* < 0.01), which is consistent with Hypothesis 1 for this variable. Moreover, we found a significant interaction between childcare coverage and education levels in the direction predicted by Hypothesis 2. In fact, Model 3 in Table 4 shows that childcare coverage and fertility are positively related for all educational groups, but this relationship is stronger for the highly educated (0.002; *p* < 0.001) and weaker for the lower-educated (−0.001; *p* < 0.1), as compared with women with a medium level of education.^6^ Again, to ease interpretations, the third graph in Fig. 2 plots the predicted number of children by educational level and childcare coverage (ranged approximately over the range of observed values). From Fig. 2, we can see that childcare coverage is positively related to fertility for all educational groups, but this association is especially substantial for highly educated women. Increasing the coverage rate from 10 % (corresponding to the observed value for Italy in 1992–1998) to 50 % (Sweden), the average number of children of low-educated women is predicted to increase from 1.8 to 2.0. For highly educated women, an equivalent increase in coverage rates leads to a jump from 1.5 to 1.9 in the predicted number of children. Interestingly, the interaction effect between education and childcare coverage makes the education gradient in fertility disappear: Significant education differences can be observed only for medium–low levels of childcare coverage.

This strong association of formal childcare with completed fertility is consistent with our expectations. By allowing a de-familialization of childcare work, childcare services facilitate both the labor market participation of women and a more gender-equal sharing of care. The strength of these results is remarkable when we consider national differences in formal childcare systems with respect to opening times, which imply that full compatibility with paid work is often not possible. Furthermore, high rates of childcare are consistent with a dual-earner model, but do not necessarily involve a gender-equal share of care work.

The relationship between standard men’s working hours and fertility shows the expected pattern. Long hours are negatively associated with the number of children, though not significantly (Table 3, Model 5). However, Model 4 in Table 4 shows a significant interaction between this variable and education. While increasing the number of men’s working hours from 38 (about the value observed for Denmark) to 45 (Greece) is associated with a small decrease in fertility for the low educated, it substantially reduces the fertility of the highly educated from 1.8 to less than 1.5 (fourth graph in Fig. 2). Long workweeks epitomize the male breadwinner model, which clearly conflicts with fertility, with the exception of the low educated. Conversely, the results suggest that policies aiming to increase fertility should take men’s time available for childcare into account.

Regarding our second indicator of working-time arrangements, the percentage of women in part-time work, our results are less clear-cut. The degree to which women have access to part-time jobs does not appear to be associated with fertility in general. But, once again, the relationship differs between education levels: The behavior of the low educated significantly differs from that of the middle and highly educated, such that part-time work is negligibly associated with fertility for the low educated. For the middle and the highly educated, an increase from 12 to 50 % in the proportion of women in part-time work is associated with a change from about 1.6 to 1.8 children per women (fifth graph in Fig. 2). However, this change is not statistically significant.

The relationship of our indicator of paid parental leave with fertility shows similar results: a positive nonsignificant overall association (Model 3, Table 3), but a significant interaction for the low educated (Model 2, Table 4). An increase in the weighted number of paid weeks of leave from 10 to 75 is associated with an increase from 1.83 to 1.92 for the low educated and to an increase from 1.56 to 1.80 for the highly educated (as in the second graph in Fig. 2).

We have considered several measures of cash transfers to families with children, including family allowances for the first, second or third child, and tax and benefit transfers for the second child. All of these indicators show a similar pattern: The overall association with fertility as well as interactions for the low and middle educated is not significant (Model 2, Table 3), while the association between these indicators and fertility for highly educated women is strongly positive (Model 1, Table 4 and first graph in Fig. 2). In principle, cash transfers should lead to an “income” effect, stimulating fertility. However, this last effect seems to be offset by a negative effect on women’s labor force participation, at least for the low and middle educated. The results suggest that this last situation would not hold for the highly educated, who most likely have a strong labor force attachment.

And what is the relationship between the prevalence of gender-egalitarian norms in the population and completed fertility? Here, our main hypothesis finds some support: The more gender-egalitarian attitudes are diffused in a country, the higher the fertility (Table 3, Model 7). Once more, the strength of the association differs sharply by education (Table 4, Model 6). Gender-egalitarian attitudes are positively related to fertility for all educational groups, but the strength of the association is stronger for women with higher education, who have the lowest probability of adhering to a traditional family model, and thus have the highest probability to gain from living in a country where gender-egalitarian attitudes are widely accepted. When the prevalence of our gender-egalitarian attitudes is 65 %, the mean predicted completed fertility is 1.4 for highly educated women and 1.75 for low-educated women. An increase in gender-equality attitudes to 95 % is associated with a convergence in fertility levels across educational groups, reaching around 2.0 children per women (sixth graph in Fig. 2).

Finally, we expected that the strength of the relationship between fertility and policies favoring the combination of work and parenthood would be stronger the more gender-equal a society is, and vice versa for policies not directed at favoring gender equality (Hypothesis 3). Overall, the results provide no evidence that gender policies are more effective if egalitarian norms are highly prevalent in the population (Table 5). This may be because in all countries egalitarian values are highly prevalent, reaching well over 50 %, suggesting that gender policies should be effective everywhere. This may also be due to the reduced number of degrees of freedom at the country level. The results also suggest that egalitarian values are independent from specific policies in enhancing fertility levels.

## Conclusions

Using data from the EU-SILC database for sixteen European countries, we have analyzed the relationship between gender policies and individual-level completed fertility. Our results provide evidence in two areas: First, policies that support particular gender relations models are associated with fertility and, second, these associations are strongly heterogeneous in the population by the level of education.

Framing the analyses in the welfare regime perspective has been useful in identifying key policies that shape gender relations and understand their relative role in the institutional context. This perspective focuses on how regulations and welfare provision affect the functioning of each institution, thus avoiding partial views restricted to welfare provision only (services or cash). Our results suggest that policies that promote de-familialization of care, via direct provision or by creating the conditions for market provision, are powerfully associated with higher fertility. A universal day care policy is likely to be a pre-condition for gender equality by reducing the total amount of care done by families and favoring a more gender-equal sharing of care and wage work.

Labor market policies that support a familialization of care offer less clear-cut results. Policy strategies centered on women’s primary care responsibility, such as part-time work, maternity leave or monetary support, show a weaker relationship with fertility. Welfare state support to family care, even if they are temporary in women’s life courses, still implies gendered costs of fertility. On the other hand, a reduction in men’s standard working hours is associated with a sharp increase in completed fertility, suggesting that working-time regulations have an important role in men’s involvement in care. Institutional contexts and policies that provide support to a dual-earner/dual-carer model are positively related to a higher fertility level, with respect to policies that maintain a preferential care role for women. These results also highlight that existing policies support a variety of gender relations, which cannot be reduced to a continuum between a male breadwinner–housewife model and a dual-earner/dual-carer model.

In trying to discern the relationship between public policies and fertility, we should pay attention to the existence of differentials in these associations by population subgroup and the mechanisms involved in explaining such differentials. Here we have hypothesized that the higher the level of gender-equality prevalent in the welfare regime institutions, the less a woman’s educational level will be negatively associated with fertility. The results of the analyses of the differential association between policies and fertility according to the level of education show theoretically consistent and statistically significant patterns. A positive association between education and fertility was found for all of the indicators analyzed, which were particularly strong for formal childcare, men’s standard working hours and gender norms. However, contrary to our expectation, we find no evidence that a high prevalence of gender-egalitarian norms in the population enhances the fertility impact of policies. This result suggests that policies and norms are closely interrelated and that no strong inconsistencies exist in the countries and cohorts studied (for instance, egalitarian policies in a context with a predominant breadwinner–housewife model). Most countries seem to be in a stage of the gender transition in which the breadwinner model has been modified to accommodate women’s labor market participation, but have not yet accommodated men’s caring role.

Overall, our findings are quite consistent with our theoretical expectations about the impact of gender policies on fertility. Of course, our data had several drawbacks, concerning in particular the suitability of the indicators available and the time lag between the measurement of the context indicators and completed fertility. The cross-sectional nature of the data used implies that no close relationship could be made between changes in polices and changes in fertility behavior. This approach involves the assumptions that our contextual variables do not change and that they apply to the moment when women (or couples) decide whether to have a(n additional) child. As we note in the section on data, the policies included in the study are highly stable for the period considered. Furthermore, our choice of analytical approach precluded us from undertaking an analysis of the timing of births and involved that no distinctions between different parities could be made. Some studies have suggested that first births are influenced by policies to a lesser extent than subsequent births (Harknett et al. 2014). Therefore, an analysis that would distinguish by parity would likely find lower relationship between policies and first births and a stronger relationship with higher parities.

Analyzing policy indicators one by one has provided clear-cut results, in particular concerning their interactions with education. However, the high correlation between policy indicators suggests caution in interpreting each of their individual effects in causal terms, since they are likely to be overestimated. An alternative empirical strategy focusing on the effects of specific constellations of variables would necessitate analyses of the situations of particular countries, which was beyond the scope of our study.

In spite of these limitations, our analyses reveal that, depending on the policy environment, fertility differentials reached about half a child, most notably when educational groups were considered. This is the order of magnitude of the existing gap between countries with very low levels and those with sustainable fertility levels. Although all educational groups seem to benefit from policies enhancing gender equality, the highly educated benefits more. The rapid increase in women’s educational levels observed in many European countries suggests a parallel increase in the demand for gender-egalitarian polices, which is consistent with economic considerations and with the higher prevalence of gender-egalitarian values among the highly educated. The diffusion of egalitarian behavior from the highly educated to the less educated could reinforce this trend. Furthermore, gender-egalitarian policies provide an explanation for the convergence in fertility levels that has been observed across educational levels in several countries. Following our arguments, this convergence can be expected to continue. As we have shown, it is a process that is fueled by policies that support the gender transition.

## Appendix

See Table 6.

**Table 6:** Correlation between country-level variables

	Family allowance	Weighted leave	Childcare coverage	Childcare usage	Men working	%Women on part-time	Gender-egalitarian norms
Family allowances	1.00						
Weighted leave	0.63	1.00					
Childcare coverage	0.04	0.03	1.00				
Childcare usage	0.20	0.34	0.83	1.00			
Men working	−0.54	−0.45	−0.58	−0.50	1.00		
%Women on part-time	0.25	−0.24	0.22	0.14	−0.42	1.00	
Gender-egalitarian norms	−0.08	0.22	0.61	0.60	−0.45	0.07	1.00

## Footnotes

1 Defamilializaton is defined as the extent to which household tasks and care work done by families is reduced by being provided from outside the household.

2 Other factors include the increase in demand for female labor, typical of postindustrial and knowledge economies, the commodification of goods and services outside the household, and the historical reduction in fertility that frees women’s time for other activities. Nevertheless, none of these factors can be considered as completely exogenous.

3 This model “was and is mainly spread in agrarian and craft families in which care was not well developed as a specific task, allocated to specific persons and needing specific skills”, Pfau-Effinger (2005, p 329).

4 We use the Vuong test to check for the presence of zero inflation. Since the test was rejected for each considered model specification, we employed the Poisson model.

5 We have to notice that the available methodological papers focussed on linear and logistic models and that there are no simulation studies specific for multilevel Poisson models.

6 Models using childcare usage instead of coverage yield very similar results. Results are available upon request.

## References

[CR1] Aassve A, Arpino B, Billari FC (2013). Age norms on leaving home: Multilevel evidence from the European Social Survey. Environment and Planning A.

[CR2] Andersson G (2000). The impact of labour-force participation on childbearing behaviour: Pro-cyclical fertility in Sweden during the 1980s and 1990s. European Journal of Population.

[CR3] Anxo, D., Flood, L., Mencarini, L., Pailhé, A., Solaz, A., & Tanturri, M. L. (2007). *Time allocation between work and family over the life*-*cycle: A comparative gender analysis of Italy, France, Sweden and the United States*. IZA Discussion Paper No. 3193, Bonn.

[CR4] Arpino B, Esping-Andersen G, Pessin L (2015). How do changes in gender role attitudes towards female employment influence fertility? A macro-level analysis. European Sociological Review.

[CR5] Arpino B, Tavares LP (2013). Fertility and values in Italy and Spain: A look at regional differences within the European context. Population Review.

[CR6] Austin, P.C. (2010). Estimating multilevel logistic regression models when the number of clusters is low: a comparison of different statistical software procedures. *The International Journal of Biostatistics*. doi:10.2202/1557-4679.1195. 10.2202/1557-4679.1195 PMC2949382 20949128

[CR7] Baizan P, Domínguez M, González MJ (2014). Couple bargaining or socio-economic status? Why some parents devote more time to their children than others. European Societies.

[CR8] Basten, S., Sobotka, T., & Zeman, K. (2013). *Future fertility in low fertility countries*. Vienna Institute of Demography Working Papers 5.

[CR9] Bernhardt E (1993). Fertility and employment. European Sociological Review.

[CR10] Bettio F, Plantenga J (2004). Comparing care regimes in Europe. Feminist Economics.

[CR11] Billingsley S, Ferrarini T (2014). Family policy and fertility intentions in 21 European countries. Journal of Marriage and Family.

[CR12] Bonoli G (2008). The impact of social policy on fertility: Evidence from Switzerland. Journal of European Social Policy.

[CR13] Bowles S (1998). Endogenous preferences: The cultural consequences of markets and other Economic institutions. Journal of Economic Literature.

[CR14] Brodmann St, Esping-Andersen G, Guell M (2007). When fertility is bargained: Second births in Denmark and Spain. European Sociological Review.

[CR15] Cipollone, A., Patacchini, E., & Vallanti, G. (2013). *Women labor market participation in Europe: Novel evidence on trends and shaping factors*. IZA Discussion Paper No. 7710.

[CR16] Coltrane S (2000). Research on household labor: Modeling and measuring the social embeddedness of routine family work. Journal of Marriage and the Family.

[CR17] Cooke LP (2004). The gendered division of labor and family outcomes in Germany. Journal of Marriage and Family.

[CR18] Crompton R, Crompton R (1999). Discussions and conclusions. Restructuring gender relations and employment: The decline of the male breadwinner.

[CR19] Del Boca D, Pasqua S, Pronzato C (2009). Motherhood and market work decisions in institutional context: A European perspective. Oxford Economic Papers.

[CR20] DiPrete ThS, Morgan Ph, Engelhardt H, Pacalova H (2003). Do crossnational differences in costs of children generate cross-national differences in fertility rates?. Population Research and Policy Review.

[CR21] Duvander A-Z, Lappegård T, Andersson G (2010). Family policy and fertility: Fathers’ and mothers’ use of parental leave and continued childbearing in Norway and Sweden. Journal of European Social Policy.

[CR22] Ermisch JF (1989). Purchased childcare, optimal family size and mother’s employment theory and econometric analysis. Journal of Population Economics.

[CR23] Esping-Andersen G (1990). The three worlds of welfare capitalism.

[CR24] Esping-Andersen G (1999). Social foundations of postindustrial economies.

[CR25] Esping-Andersen G (2009). The incomplete revolution: Adapting to women’s new roles.

[CR26] Esping-Andersen G, Billari FC (2015). Re-theorizing family demographics. Population and Development Review.

[CR27] Fent T, Aparicio Diaz B, Prskawetz A (2013). Family policies in the context of low fertility and social structure. Demographic Research.

[CR28] Folbre N (1994). Who pays for the kids? Gender and the structures of constraint.

[CR29] Gauthier AH (2007). The impact of family policies on fertility in industrialized countries: A review of the literature. Population Research and Policy Review.

[CR30] Gauthier, A. H. (2011). *Comparative family policy database, version 3 [computer file]. Netherlands Interdisciplinary Demographic Institute and Max Planck Institute for Demographic Research (distributors)*. Retrieved from www.demogr.mpg.de

[CR31] Gauthier AH, Hatzius J (1997). Family benefits and fertility: An econometric analysis. Population Studies.

[CR32] Goldscheider F, Bernhardt E, Brandén M (2013). Domestic gender equality and childbearing in Sweden. Demographic Research.

[CR33] Goldstein H, Healy MJR (1995). The graphical presentation of a collection of means. Journal of the Royal Statistical Society, A.

[CR34] González, M. J., Jurado-Guerrero, T., & Naldini, M. (2009). *What made him change? An individual and national analysis of men’s participation in housework in 26 countries*. DemoSoc Working Paper 30.

[CR35] Gornick JC, Meyers M (2003). Families that work: Policies for reconciling parenthood and employment.

[CR36] Gustafsson S, Stafford F, Blank R (1994). Three regimes of childcare: The United States, the Netherlands, and Sweden. Social protection versus economic flexibility.

[CR37] Hakim C (2000). Work-lifestyle choices in the 21st century: Preference theory.

[CR38] Harknett K, Billari FC, Medalia C (2014). Do family support environments influence fertility? Evidence from 20 European countries. European Journal of Population.

[CR39] Hoem JM (2008). The impact of public policies on European fertility. Demographic Research.

[CR40] Hook JL (2010). Gender inequality in the welfare state: Sex segregation in housework, 1965–2003. American Journal of Sociology.

[CR41] Hotz VJ, Klerman JA, Willis RJ, Rosenzweig MR, Stark O (1997). The economics of fertility in developed countries. Handbook of population and family economics.

[CR42] Human Fertility Database (HFD). (2014). *Max Planck Institute for Demographic Research (Germany) and Vienna Institute of Demography (Austria)*. www.humanfertility.org. Accessed August 2014.

[CR43] Jakee, K., & Sun, G.-Z. (2001). *Adaptive preferences and welfare state dynamics: A simple model. Max*-*Planck*-*Institute for Research into economic systems (Jena, Germany)*. Papers on Economics and Evolution, 2.

[CR44] Joshi H (1998). The opportunity costs of childbearing: More than mothers’ business. Journal of Population Economics.

[CR45] Kan MY, Sullivan O, Gershuny J (2011). Gender convergence in domestic work: Discerning the effects of interactional and institutional barriers from large-scale data. Sociology.

[CR100] Keck, W., & Saraceno, C. (2013). The impact of different social-policy frameworks on social inequalities among women in the European Union: The labour-market participation of mothers. *Social Politics*, *20*(3), 297–328.

[CR46] Keck, W., Hessel, P., & Saraceno, C. (2009). *Database on intergenerational policy indicators: Methodological report*. Berlin: Wissenschaftszetrum Berlin für Sozialforschung/Rotterdam: Multilinks project.

[CR47] Korpi W (2000). Faces of inequality: Gender, class and patterns of inequalities in different types of welfare states. Social Politics.

[CR48] Korpi W (2000). Class and gender inequalities in different types of welfare states: The Social Citizenship Indicator Program (SCIP). International Journal of Social Welfare.

[CR49] Korpi W, Ferrarini T, Englund S (2013). Women’s opportunities under different family policy constellations: Gender, class, and inequality tradeoffs in western countries re-examined. Social Politics.

[CR50] Kravdal O, Rindfuss RR (2008). Changing relationships between education and fertility: A study of women and men born 1940 to 1964. American Sociological Review.

[CR51] Lewis J (1992). Gender and the development of welfare regimes. Journal of European Social Policy.

[CR52] Lewis J (1998). Gender, social care and welfare state restructuring in Europe.

[CR53] Lewis J (2002). Gender and welfare state change. European Societies.

[CR54] Mason K Oppenheim, Jones GW (1997). Gender and demographic change: What do we know?. The continuing demographic transition.

[CR55] Mayer KU, Woodward A, Kohli M (2001). The paradox of global social change and national path dependencies. Life course patterns in advanced societies. Inclusions and exclusions in European societies.

[CR56] McDonald P (2000). Gender equity, social institutions and the future of fertility. Journal of Population Research.

[CR57] McDonald P (2000). Gender equity in theories of fertility transition. Population and Development Review.

[CR58] McDonald P (2006). Low fertility and the state: The efficacy of policy. Population and Development Review.

[CR59] McNeish, D. M., & Stapleton, L. M. (2014). The effect of small sample size on two-level model estimates: A review and illustration. *Educational Psychology Review*. doi:10.1007/s10648-014-9287-x.

[CR60] McNicoll, G. (1994). *Institutional analysis of fertility*. The Population Council Research Division Working Paper 72.

[CR61] Mencarini L, Tanturri ML (2004). Time use, family role-set and childbearing among Italian working women. Genus.

[CR62] Mills M (2010). Gender roles, gender (in)equality and fertility: An empirical test of five gender equity indices. Canadian Studies in Population.

[CR63] Mills M, Menacarini L, Tanturri ML, Begall K (2008). Gender equity and fertility intentions in Italy and the Netherlands. Demographic Research.

[CR64] Myrskyla, M., Billari F. C., & Kohler H-P. (2011). *High development and fertility: Fertility at older reproductive ages and gender equality explain the positive link*. MPIDR Working Papers WP-2011-017, Max Planck Institute for Demographic Research, Rostock, Germany.

[CR65] Neyer G, Andersson G (2008). Consequences of family policies on childbearing behavior: Effects or artifacts?. Population and Development Review.

[CR66] Neyer G, Lappegard T, Vignoli D (2013). Gender equality and fertility: Which equality matters?. European Journal of Population.

[CR67] OECD. (2014). *OECD stat extracts*. http://stats.oecd.org/Index.aspx. Accessed July 2014.

[CR68] Oláh L Sz (2003). Gendering fertility: Second births in Sweden and Hungary. Population Research and Policy Review.

[CR69] Orloff AS (1993). Gender and social rights of citizenship: The comparative analysis of gender relations and welfare states. American Sociological Review.

[CR70] Orloff, A. S. (2002).*Women’s employment and welfare regimes globalization, export orientation and social policy in Europe and North America*. Social Policy and Development Programme, Paper Number 12. Geneva: United Nations Research Institute for Social Development.

[CR71] Paccagnella O (2011). Sample size and accuracy of estimates in multilevel models: New simulation results. Methodology: European Journal of Research Methods for the Behavioral and Social Sciences.

[CR72] Pettit B, Hook J (2009). Institutionalizing inequality: Gender, family, and economic inequality in comparative perspective.

[CR73] Pfau-Effinger B (2004). Development of culture, welfare States and women’s employment in Europe.

[CR74] Pfau-Effinger B (2005). Welfare state policies and the development of care arrangements. European Societies.

[CR75] Plantenga J, Remery C, Siegel M, Sementini L, Leira A, Saraceno C (2008). Childcare Services in 25 European Union Member States: The Barcelona targets revisited. Childhood: Changing contexts.

[CR76] Ray R, Gornick JC, Schmitt J (2010). Who cares? Assessing generosity and gender equality in parental leave policy designs in 21 countries. Journal of European Social Policy.

[CR77] Rindfuss RR, Brewster KL (1996). Childrearing and fertility. Population and Development Review.

[CR78] Rubery J, Smith M, Fagan C (1998). National working-time regimes and equal opportunities. Feminist Economics.

[CR79] Saraceno, C., & Keck, W. (2009). *The institutional framework of intergenerational family obligations in Europe: A conceptual and methodological overview*. Report of the Multilinks Project.

[CR80] Saraceno C, Keck W (2013). The impact of different social-policy frameworks on social inequalities among women in the European Union: The labour-market participation of mothers. Social Politics.

[CR81] Seguino S (2007). Plus Ça change? Evidence on global trends in gender norms and stereotypes. Feminist Economics.

[CR82] Shang Q, Weinberg BA (2013). Opting for families: Recent trends in the fertility of highly educated women. Journal of Population Economics.

[CR83] Sigle-Rushton, W., & Waldfogel, J. (2004). *Family gaps in income: A cross*-*national comparison*. Maxwell School of Citizenship and Public Affairs Working Paper 382.

[CR84] Skrondal A, Rabe-Hesketh S (2004). Generalized latent variable modeling: Multilevel, longitudinal, and structural equation models.

[CR85] Thévenon O, Gauthier A (2011). Family policies in developed countries: A fertility booster with side-effects. Community Work and Family.

[CR86] Torr BM, Short S (2004). Second births and the second shift: A research note on gender equity and fertility. Population and Development Review.

[CR87] Waldfogel J, Han W-J, Brooks-Gunn J (2002). The effects of early maternal employment on child development. Demography.

[CR88] Wall, K. (2007). Leave policy models and the articulation of work and family in Europe. In P. Moss & K. Wall (Eds.), *International review of leave policies and related research* (pp. 25–43). Employment relations research series no. 80. London: BERR.

[CR89] Williams J (2000). Unbending gender.

